# Inflammatory parameters as predictive factors for complicated appendicitis: A retrospective cohort study

**DOI:** 10.1016/j.amsu.2022.103266

**Published:** 2022-01-19

**Authors:** Ana Matos Ribeiro, Inês Romero, Carlos Costa Pereira, Filomena Soares, Álvaro Gonçalves, Susana Costa, João Barros da Silva

**Affiliations:** a Centro Hospitalar Tâmega e Sousa, Penafiel, Portugal; b Hospital de Braga, Braga, Portugal

**Keywords:** Ileocecal appendix, Acute appendicitis, Predictive factors, Leukocytosis, C-reactive protein, Ratio between neutrophils and lymphocytes

## Abstract

**Introduction:**

Acute appendicitis is a major cause of acute abdomen. Although its diagnosis is clinical, it is often supported by complementary diagnostic tests. Sometimes, delay in diagnosis can lead to worsening of the clinical picture, resulting in a complicated acute appendicitis. Some series have studied some clinical and analytical parameters as possible predictors of complicated acute appendicitis.

**Study design:**

A retrospective analysis of patients admitted for acute appendicitis and undergoing appendectomy between January 2014 and December 2017 was performed in order to assess the possible existence of preoperative analytical predictive factors for complicated acute appendicitis (such as leukocytosis, C-reactive protein and ratio between neutrophils and lymphocytes).

**Results:**

841 patients underwent emergency appendectomy during the analysed period. This initial sample was divided into two groups: Group 1 with patients with uncomplicated acute appendicitis and Group 2 with patients with complicated acute appendicitis. Group 2's presentation age, duration of symptoms and hospital stay was significantly higher than Group 1. Regarding analytical parameters, the measurement of leukocytes, C-reactive protein and ratio between neutrophils and lymphocytes was significantly higher in patients with complicated acute appendicitis. After a multivariate analysis, it was found that only C-reactive protein was a good predictor of complicated acute appendicitis.

**Conclusion:**

Several publications have studied and demonstrated the possible use of certain analytical parameters as predictors of complicated acute appendicitis. In our study, C-reactive protein proved to be a good independent predictor of complicated acute appendicitis and, therefore, when an assay of this protein exceeds 63.3 mg/L, faster surgical approach should be considered due to the high probability of the presence of a complicated picture of this clinical entity.

## Highlights

• Preoperative distinction of the severity of appendicitis can be challenging.
• Biochemical markers can help the surgeon to understand its severity and emergency.
• C-reactive protein shows to be a good predictor of complicated acute appendicitis.

## 1 Introduction

Acute appendicitis is an inflammatory process of the ileocecal appendix. It is one of the most common causes of acute non-traumatic abdomen and subsequent appendectomy is one of the most urgent surgical procedures performed [1,2]. It typically presents as abdominal pain located in the right iliac fossa, with a few hours of evolution, often preceded by peri-umbilical pain due to the stimulation of visceral afferent fibers [1].

The diagnosis is essentially clinical and supported by complementary diagnostic tests if necessary (leucogram, C-reactive protein, ultrasound, computed tomography). When this entity is diagnosed early and appendectomy is performed in a timely manner, the prognosis is favourable [1]. There are potential complications from an inflammatory process at the ileocecal appendix. Acute peritonitis due to perforated appendix and the formation of abscess are seen in some patients [3].

Several authors have studied some possible predictive factors (C-reactive protein, leukocytosis, duration of symptoms, presence of fever) of complicated appendicitis to evaluate the urgency of the surgical approach, in order to help distinguish the severity of this pathology [2,3,4,5,6].

In this article, a retrospective study is presented to assess the existence of possible analytical predictive factors for complicated acute appendicitis, in the preoperative phase.

## 2 Methods

In this study, a retrospective analysis was performed of all patients admitted for acute appendicitis at our Hospital, between January 2014 and December 2017. No ethical approval was necessary to obtain as it is a retrospective study. This study was retrospectively registered in ClinicalTrials.gov, with the registration number NCT05116124 (https://clinicaltrials.gov/ct2/show/NCT05116124?id=NCT05116124&draw=2&rank=1). Only cases of uncomplicated and complicated acute appendicitis submitted to surgery were included, posteriorly confirmed by anatomopathological examination. A sample of 841 patients was obtained.

Based on the patients' clinical records, some variables were evaluated, such as age, gender, duration of symptoms presented and length of hospital stay. In addition, assays for the following inflammatory parameters were also analysed: leukocytes, C-reactive protein (CRP) and the ratio between neutrophils and lymphocytes. Regarding the surgical technique, laparoscopic or open appendectomy was performed. Based on surgical and pathological findings, acute appendicitis was classified as uncomplicated appendicitis or complicated appendicitis. Cases of complicated acute appendicitis were defined as appendicitis with abscess or free perforation. The study's sample was divided into two groups: a first group (Group 1) classified as uncomplicated appendicitis and a second group (Group 2) classified as complicated appendicitis.

In our study, for statistical analysis, a logistic regression analysis of SPPS ver. 26.0 (Statistical Package for the Social Sciences version 26.0) was applied. Categorical variables are presented as frequencies and percentages, and continuous variables as medians and interquartile ranges for variables with skewed distributions. Normal distribution was checked using Shapiro-Wilk test. Multivariate analysis was performed using logistic regression. The cut-off value for each of the inflammatory parameters evaluated was defined using the Receiver Operating Characteristic Curve (ROC curve), relating to sensitivity and specificity and obtaining the highest Youden index. A p value < 0.05 was considered to be statistically significant. This article has been reported in line with the STROCSS 2021 criteria [7].

## 3 Results

Of the 841 patients obtained in the initial sample, there were 448 men (53.3%) and 393 women (46.7%). This initial sample was divided into two groups, according to the severity presented. The Group 1 was classified as uncomplicated appendicitis and the Group 2 was classified as complicated appendicitis. Patients were divided between both groups, with Group 1 consisting of 564 patients (67.1%) and Group 2 of 277 patients (32.9%) with acute appendicitis undergoing appendectomy.

The overall median age was 38 years old [age range between 18 and 92 years old; with an interquartile range (IQR) of 27] (Table 1). Group 1 presented a median age of 35 years old (18–89 years old; IQR 23), while Group 2 had a significantly higher median (p < 0.001), of 48 years old (18–92 years old; IQR 31) (Table 2).

**Table 1:** General characteristics of the initial sample.

Variables	Initial sample (n = 841)
Age (years)	38 (IQR 27)
Symptoms duration (days)	1 (IQR 1)
Hospital stay (days)	3 (IQR 2)
Leukocytosis (^10^3^/μL)	14.1 (IQR 5.25)
CRP (mg/L)	45.5 (IQR 98.5)
Neutrophils/lymphocytes	7.21 (IQR 6.72)

IQR, interquartile range; CRP, C-reactive protein.

**Table 2:** General characteristics and univariate analysis according to appendicitis’ severity.

Variables	Uncomplicated (n = 564)	Complicated (n = 277)	*p* value
Age (years)	35 (IQR 23)	48 (IQR 31)	<0.001
Symptoms duration (days)	1 (IQR 1)	2 (IQR 2)	<0.001
Hospital stay (days)	2 (IQR 1)	5 (IQR 4)	<0.001
Leukocytosis (^10^3^/μL)	13.85 (IQR 4.88)	14.5 (IQR 6.2)	0.004
CRP (mg/L)	32 (IQR 55.68)	115.1 (IQR 153)	<0.001
Neutrophils/lymphocytes	6.27 (IQR 5.6)	9.52 (IQR 8.64)	<0.001

IQR, interquartile range; CRP, C-reactive protein.

The median duration of symptoms of the initial sample was 1 day (IQR 1). This was higher in the complicated appendicitis group, with a median of 2 days, compared to the median of 1 day in the uncomplicated appendicitis group (p < 0.001). In general, the median length of hospital stay was 3 days (IQR 2), having been significantly longer in patients with complicated appendicitis (5 days, p < 0.001), when compared with Group 1 with a median length stay of 2 days (IQR 1).

All patients included in this study underwent an analytical study when admitted to the emergency department, obtaining the preoperative measurement of the analytical parameters.

The overall median leukocytosis was 14.1^10^3^/μL (IQR 5.25), and patients with complicated appendicitis had a median leukocytosis of 14.5^10^3^/μL (IQR 6.2), significantly higher (p = 0.004) than the median of 13.85^10^3^/μL (IQR 4.88) presented by uncomplicated appendicitis group.

The overall median of C-reactive protein was 45.5 mg/L (IQR 98.5), with Group 1 presenting a median of 32 mg/L and Group 2 a median of 115.1 mg/L (IQR 153), with a significantly higher difference in this Group 2 (p < 0.001).

The median of the ratio between neutrophils and lymphocytes in all patients was 7.21 (IQR 6.72). This ratio also showed a statistically significant difference (p < 0.001), with the complicated appendicitis group having a higher ratio of 9.52 (IQR 8.64).

In order to assess whether these inflammatory parameters would be good predictors of complicated disease, a multivariate analysis of all these analytical variables was performed. With this multivariate analysis (Table 3), both CRP and ratio between neutrophils and lymphocytes appear to be potential predictors of complicated appendicitis because they present statistically significant values (both with p < 0.0001), in contrast to leukocytes (p = 0.005).

**Table 3:** Results of multivariate analysis.

Variables	Confidence interval 95%	*p* value
Leukocytes	0.527–0.595	0.005
CRP	0.747–0.804	<0.0001
Neutrophils/lymphocytes	0.649–0.713	<0.0001

CRP, C-reactive protein.

**Fig. 1:**
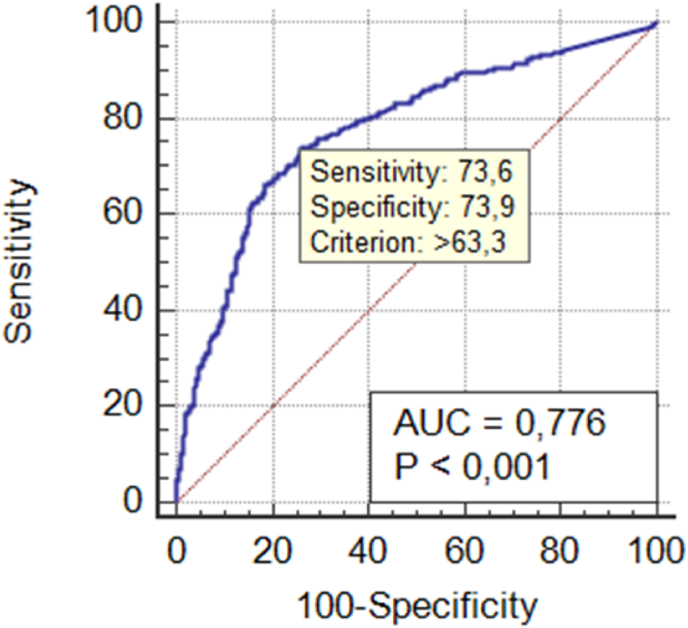
C-reactive protein ROC curve.

**Fig. 2:**
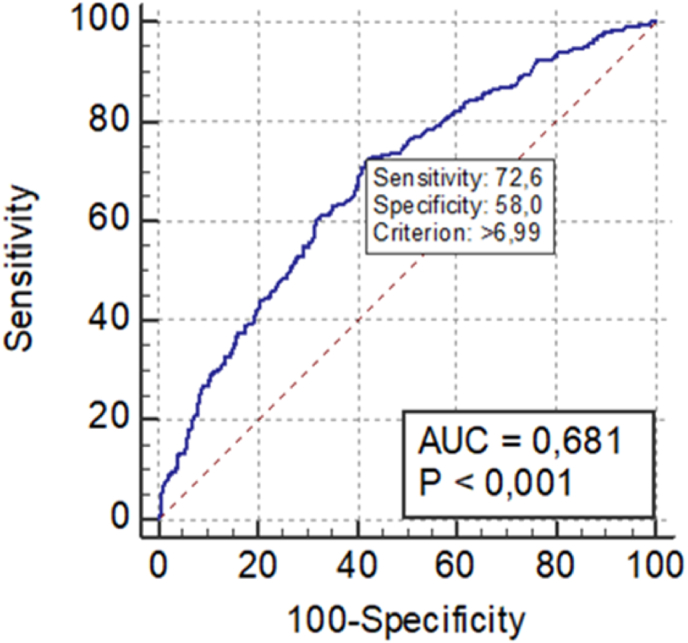
Ratio between neutrophils and lymphocytes ROC curve.

When analysing the ROC curves, the area under the C-reactive protein curve was 0.776 (Fig. 1) and the one of the ratio between neutrophils and lymphocytes was 0.681 (Fig. 2). In order to consider a good ROC curve area, it needs to have a value equal or greater than 0.700. Therefore, only CRP proved to be a good predictor of complicated acute appendicitis, in contrast to the other inflammatory parameters.

The highest value of the Youden index was obtained when applying sensitivity and specificity. When the CRP value was 63.3 mg/L, the highest Youden index was 0.476, with a sensitivity of 73.65% and a specificity of 73.94%. Besides, it demonstrated a positive predictive value of 58.1% and a negative predictive value of 85.1%, so there is a probability that about 85% of patients with a CRP value of less than 63.3 mg/L will not actually have a complicated acute appendicitis.

## 4 Discussion

Acute appendicitis is a major cause of acute abdomen, making appendectomy one of the most commonly performed surgeries, either by laparoscopy or laparotomy. Despite its high incidence, some studies report a misdiagnosis rate between 12 and 30%. Sometimes, acute appendicitis becomes a more complicated condition, especially in children and in elderly, due to a less typical clinical presentation, making its diagnosis more difficult and with greater potential to mimic other pathologies. Some studies suggest a higher incidence of complicated appendicitis in patients older than 40 years old [2,[8], [9], [10]]. In our study, patients were also significantly older in the complicated disease group.

Another important finding in our study is that patients who had a more severe condition took longer to seek medical help, with a significantly longer duration of symptoms (2 days), compared to patients with uncomplicated appendicitis. The incidence of contained or free perforation associated with appendicitis is superior when there is a delay in its diagnosis, whether patient-related or physician-related, these findings being confirmed by previous studies [6,11,12].

The preoperative distinction between complicated and uncomplicated appendicitis can be challenging, except when in the presence of evident signs of peritoneal irritation or through the use of imaging studies, such as ultrasound or computed tomography, which may show signs of a complicated condition (such as intrabdominal abscesses, pneumoperitoneum or free intrabdominal liquid). However, the use of these studies is not strictly necessary or always performed for the diagnosis of acute appendicitis, since its diagnosis is clinical [1,3]. The distinction between uncomplicated or complicated condition does not change therapeutic approach, but it helps the surgeon to understand the severity and the emergency with which he may have to act.

Although some studies have discussed several factors associated with the diagnosis of acute appendicitis, definitive preoperative diagnosis of complicated appendicitis based on preoperative factors is difficult. For this reason, several authors have studied the importance and possible existence of certain findings, essentially clinical or biochemical ones, that can help distinguish the severity of this major cause of acute abdomen. However, there are few published studies on this matter [3,6,13].

In order to identify preoperative predictive factors that may help to distinguish the severity of acute appendicitis, a retrospective study to determine the validity of three potential factors was conducted (leukocytes, C-reactive protein and ratio between neutrophils and lymphocytes). Some studies have showed the possible importance of these analytical parameters in the diagnosis of acute appendicitis.

Leukocytes are pluripotent hematopoietic stem cells in the bone marrow, which migrate to inflammatory sites and where they are activated in order to secrete substances such as cytokines, complement components, growth factors, proteases and nitric oxide, substances that are primary sources of tissue damage, involved in inflammatory and infectious states [14,15].

Leukocyte assay has been used for the diagnosis of acute appendicitis, and even some authors have studied the possibility of differentiating complicated acute appendicitis based on certain leukocytes values. Some studies have reported a median of leukocytosis between 12.9 × 10^3^/μL and 14.6 × 10^3^/μL in patients with uncomplicated acute appendicitis and a median between 13.3 × 10^3^/μL and 17.4 × 10^3^/μL in patients with complicated acute appendicitis [13,16]. Our median of leukocytosis in both groups (13.85 × 10^3^/μL in Group 1 and 14.5 × 10^3^/μL in Group 2) is similar to the median found in the literature. Leukocytosis has shown in literature a sensitivity ranging from 79 to 93% in cases of acute appendicitis, however with low specificity. As in our study, Sack U et al. didn't also demonstrate an association between a certain value of leukocytosis and complicated acute appendicitis patients, and has not been validated as a good predictor [17].

Neutrophils and lymphocytes are leukocyte blood cells involved in the innate immune system. Under physiological stress, such as inflammatory or infectious situations, the number of neutrophils rises while the number of lymphocytes decreases. The ratio between these cells increases more rapidly after this physiological stress then other laboratory parameters, such as leukocytes. Besides, even with normal values of leukocytes, this ratio has been shown to be a predictor of inflammatory processes [[18], [19], [20]]. To the extent of our knowledge, no other study has been published on the possible use of this ratio to help distinguish the severity of acute appendicitis. Although our study demonstrated a significantly higher ratio between neutrophils and lymphocytes in patients with complicated acute appendicitis (9.52 versus 6.27; p < 0.001), this analytical parameter did not prove to be a good predictor of a more severe condition.

C-reactive protein is an acute inflammatory protein that rises at sites of inflammation or infection, accelerating phagocytic reactions, chemotaxis and platelet activation and increasing cell-mediated immunity. This protein is then used as a marker of acute disease, such as in acute appendicitis [21,22].

The first study found to report an association between increased levels of C-reactive protein and acute appendicitis was carried out by Mikaelsson C and Arnbjornsson E in 1984. This study demonstrated a normal value of this protein in patients with an ileocecal appendix without inflammatory changes and an increased value in patients with acute appendicitis, this value being even higher in situations of more severe inflammation [23]. In fact, in 1994, Albu E et al. reported that in patients with symptoms duration for at least 12 h and with a CRP dose below 25 mg/L, acute appendicitis could be excluded [24]. More recently, has been described in the literature a median of C-reactive protein in complicated appendicitis ranging from 97.6 mg/L to 101 mg/L [13]. In our study, a significantly higher dosage was noted in these patients, with a value higher than that reported in literature (115.1 mg/L).

Several studies report a sensitivity of C-reactive protein between 40 and 88% and a specificity between 46 and 98.3%, values that are compatible with the findings in our study (sensitivity of 73.65% and specificity of 73.94%). Moon HM et al. demonstrated that the cut-off value of CRP (with the highest Youden index) was 70.5 mg/L, with a sensitivity of 57.6% and a specificity of 98.3% (with positive predictive value of 97.4% and negative predictive value of 68.5%) [12,13,17,25,26]. This cut-off value is very similar to the one found in our study (63.3 mg/L), however with a higher sensitivity and negative predictive value (73.65% and 85.1%, respectively), very close to the maximum values described in literature.

Among the analytical parameters evaluated in our study, C-reactive protein is the only one that proved to be a good predictor of complicated acute appendicitis. Sack U et al. also presented CRP as a good predictor of the severity of this clinical entity [17].

To the extent of our knowledge, no other published study has presented a sample of patients with acute appendicitis with a number as high as ours, nor has it concomitantly evaluated these three analytical inflammatory parameters, which frequently, quickly and easily, are or can be measured in patients admitted to the emergency department with abdominal pain suspected of acute appendicitis. In addition, also to our knowledge, this is the first study to mention the possible association between certain values of the ratio between neutrophils and lymphocytes and a complicated case of acute appendicitis.

The conclusions of our study are limited due to the fact that it is a retrospective study and based on a single hospital, despite presenting a good sample size of patients with acute appendicitis. Therefore, it is essential to develop prospective studies in order to confirm the possibility of diagnosing or even excluding a complicated acute appendicitis through determination of certain values of C-reactive protein, or even other analytical or clinical parameters. However, it is noteworthy that the discrimination of cases based only on one or two predictive factors is controversial, requiring further studies, mainly prospective and randomized, to support decisions regarding the performance of emergent or urgent surgery.

## 5 Conclusion

The incidence of complicated appendicitis is still considerable, especially in extremes of age, often due to atypical presentation symptoms, making diagnosis more difficult. C-reactive protein is one of the most used markers of acute inflammation, easily accessible and with quick results, when an inflammatory or infectious condition is suspected. Several published studies have shown an association between higher values of this protein and the presence of complicated appendicitis. In these studies, its sensitivity for the diagnosis of complicated appendicitis varied between 40 and 87%, with a specificity ranging from 53 to 82%. In our study, CRP presented values of sensitivity and specificity close to the maximum value described in literature. We conclude from our data that C-reactive protein seems to be an independent predictor of complicated acute appendicitis and, therefore, when its measurement is above 63.3 mg/L, an early approach should be considered due to the high probability of complicated appendicitis.

## Ethical approval

No ethical approval was obtained as it is a retrospective study.

## Sources of funding

This research did not receive any specific grant from funding agencies in the public, commercial, or not-for-profit sectors.

## Author contributions

Ana Matos Ribeiro – Study design, data collection, statistical analysis, writing the paper.

Inês Romero – Study design, reviewing the paper.

Carlos Costa Pereira – Study design, reviewing the paper.

Filomena Soares – reviewing the paper.

Álvaro Gonçalves – reviewing the paper.

Susana Costa – reviewing the paper.

João Barros da Silva – reviewing the paper.

## Research registration number

1. Registry used: ClinicalTrials.gov
2. Unique Identifying number or registration ID: NCT05116124
3. Hyperlink to your specific registration (must be publicly accessible and will be checked) https://clinicaltrials.gov/ct2/show/NCT05116124?id=NCT05116124&draw=2&rank=1.

## Guarantor

Ana Matos Ribeiro.

## Provenance and peer review

Not commissioned, externally peer-reviewed.

## Declaration of competing interest

All authors have nothing to disclose.
